# Fatigue mitigation with SleepTrackTXT2 in air medical emergency care systems: study protocol for a randomized controlled trial

**DOI:** 10.1186/s13063-017-1999-z

**Published:** 2017-06-05

**Authors:** P. Daniel Patterson, Charity G. Moore, Frank X. Guyette, Jack M. Doman, Denisse Sequeira, Howard A. Werman, Doug Swanson, David Hostler, Joshua Lynch, Lindsey Russo, Linda Hines, Karen Swecker, Michael S. Runyon, Daniel J. Buysse

**Affiliations:** 1 Department of Emergency Medicine, University of Pittsburgh, School of Medicine, 3600 Forbes Avenue, Iroquois Bldg, Suite 400A, Pittsburgh, PA 15260 USA; 2 Carolinas HealthCare System, Charlotte, NC USA; 3 MedFlight of Ohio, Columbus, OH USA; 4 MedCenter Air, Department of Emergency Medicine, Carolinas HealthCare System, Charlotte, NC USA; 5 Department of Exercise and Nutrition Sciences, The State University of New York, University at Buffalo, Buffalo, NY USA; 6 Department of Psychiatry, University of Pittsburgh, School of Medicine, Pittsburgh, PA USA; 7 MercyFlight of Western New York, Buffalo, NY USA; 8 Jacobs School of Medicine and Biomedical Sciences, University at Buffalo, Buffalo, NY USA; 9 Department of Emergency Medicine, Carolinas HealthCare System, Charlotte, NC USA

**Keywords:** Shiftwork, Sleepiness, Fatigue, Alertness, Emergency medicine, Randomized controlled trial

## Abstract

**Background:**

Most air medical Emergency Medical Services (EMS) clinicians work extended duration shifts, and more than 50% report inadequate sleep, poor sleep quality, and/or poor recovery between shifts. The SleepTrackTXT pilot trial (ClinicalTrials.gov, NCT02063737) showed that use of mobile phone text messages could impact EMS clinician self-reported fatigue and sleepiness during long duration shifts. The purpose of the SleepTrackTXT2 trial is to leverage lessons learned from the first SleepTrackTXT study and test an enhanced intervention targeting air medical EMS clinicians.

**Methods/design:**

We will conduct a multi-site randomized trial with a sample of adult EMS clinicians recruited from four air medical EMS systems located in the midwest, northeastern, and southern USA. Participants will be allocated to one of two possible arms for a 4-month (120-day) study period. The intervention arm will involve text-message assessments of sleepiness, fatigue, and difficulty concentrating at the beginning, every 4 hours during, and at the end of scheduled shifts. Participants reporting high levels of sleepiness, fatigue, or difficulty with concentration will receive one of nine randomly selected intervention messages to promote behavior change during shift work to improve alertness. Intervention participants will receive a text-message report on Friday of each week that shows their sleep debt over the previous 7 days followed by a text message to promote paying back sleep debt recovery when feasible. Participants in the control group receive text messages that only include assessments. Both arms will receive text-message assessments of perceived recovery since last shift, sleepiness, fatigue, or difficulty with concentration at noon (1200 hours) on days between scheduled shifts (off-duty days). We have two aims for this study: (1) to determine the short-term impact of the enhanced SleepTrackTXT2 intervention on air medical clinician fatigue reported in real time during and at the end of shift work, and (2) to determine the long-term impact of the SleepTrackTXT2 intervention on sleep quality and sleep health indicators including hours of sleep and recovery between shift work.

**Discussion:**

The SleepTrackTXT2 trial may provide evidence of real-world effectiveness that would support widespread expansion of fatigue mitigation interventions in emergency care clinician shift workers. The trial may specifically support use of real-time assessments and interventions delivered via mobile technology such as text messaging.

**Trial registration:**

ClinicalTrials.gov, NCT02783027. Registered on 23 May 2016.

**Electronic supplementary material:**

The online version of this article (doi:10.1186/s13063-017-1999-z) contains supplementary material, which is available to authorized users.

## Background

Clinicians who work in the Emergency Medical Services (EMS) setting are shift workers who often work 12 or 24 hours in a row with periods of heightened stress and emergency clinical care separated by variable periods of rest. Shift work, i.e., work performed outside of normal daylight hours, has been linked to sleep deprivation, fatigue, and negative safety outcomes for patients and clinicians in numerous healthcare settings [1–4]. Half of EMS clinicians obtain less than 6 hours of sleep per day, more than half report poor sleep quality and fatigue, and half report poor recovery between scheduled shifts [5–8]. Few studies have tested interventions designed to mitigate fatigue and improve sleep of EMS clinician shift workers [7, 9].

Fatigue is described as “a subjective, unpleasant symptom, which incorporates total body feelings ranging from tiredness to exhaustion creating an unrelenting overall condition which interferes with an individual’s ability to function to their normal capacity” [10]. Fatigue has been linked to EMS clinician workplace injury, medical errors, adverse patient events, and behaviors that compromise the safety of patients and clinicians [6]. Reports of fatigue-related ambulance crashes and other adverse events involving EMS clinicians are on the rise [11–13]. In 2013, the National EMS Advisory Council (a US federally recognized advisory council) charged government officials with addressing the problem of fatigue in EMS and providing guidance to the EMS community to aid in fatigue management [14]. Recently, the US Department of Transportation, National Highway Traffic Safety Administration funded a project to review the evidence on fatigue risk management for purposes of creating evidence-based recommendations for fatigue risk management of EMS clinicians (www.emsfatigue.org).

Fatigue was the target of our recently completed 90-day, two-arm, parallel group, single-blind randomized trial targeting EMS clinicians (the SleepTrackTXT trial: ClinicalTrials.gov NCT02063737). The trial demonstrated the feasibility of using mobile phone text messages to perform real-time assessment and intervention of EMS clinicians who report fatigue and sleepiness during shift work [7, 9]. Attrition was low (15%), compliance with repeated assessments of fatigue and sleepiness during shift work was high (88%), and participants in the intervention group reported lower levels of fatigue and sleepiness during select time points of extended duration shifts compared to controls [7]. Improvement in self-rated sleep quality from baseline to 90 days was statistically significant for the intervention group, but not clinically meaningful [7]. Enhancement of the SleepTrackTXT intervention may lead to greater impact on sleep quality, fatigue, sleepiness during shift work, and other components of sleep health such as inter-shift recovery.

In this paper, we present a new study protocol for a 120-day (4-month) two-arm, parallel, single-blind controlled trial with an assessment-only control group modeled after the SleepTrackTXT trial [7, 9]. Our study has two aims. First, we aim to determine the short-term impact of the enhanced SleepTrackTXT2 intervention on air medical clinician fatigue reported in real time during and at the end of shift work. Second, we aim to determine the longer term impact of the SleepTrackTXT2 intervention on sleep quality and other sleep health indicators including hours of sleep and recovery between shifts. Findings will also provide important data to address more in-depth questions related to use of mobile technology and content of intervention materials specific to fatigue risk management of EMS clinicians.

## Methods/design

### Trial design

**Fig. 1:**
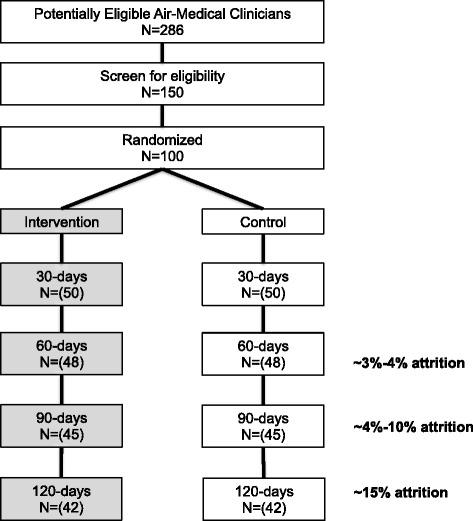
Flowchart of the SleepTrackTXT2 trial

The SleepTrackTXT2 study is a multi-site, two-arm, parallel group, randomized, controlled, single-blind trial (participants blinded). Recruitment involves circulating a study flyer to air medical clinicians employed at four air medical EMS systems in the midwest, northeastern, and southern regions of the USA. Clinicians will review the flyer for study information and eligibility requirements. Persons interested in participating are instructed to contact study coordinators and undergo screening. We will randomize 100 air medical clinicians to one of two groups (*n* = 50 each). The details of our study are described in accordance with the Standard Protocol Items: Recommendations for Interventional Trials (SPIRIT) statement. Figure 1 is a flowchart of our trial depicting the target enrollment and projected rates of attrition. The SPIRIT checklist is provided in Additional file 1. Our study protocol was approved by the Institutional Review Boards for each study site with one site, the University of Pittsburgh, serving as the study’s coordinating center and adjudicator of endpoints. The study was registered at ClinicalTrials.gov (NCT02783027) on 23 May 2016. The study’s first participant was enrolled on 6 June 2016.

### Setting

This is a multi-site study of four air medical EMS systems located in three of the four major US Census regions (midwest, northeast, and south). Study site 1 is a critical care EMS system that provides air- and ground-based emergency services across four states in the northeastern USA. The system’s patient volume exceeds 10,000 annually. The site employs more than 160 air medical clinicians who mostly work 24-hour shifts, with some 12-hour shifts at select base sites. Study site 2 provides ground-based as well as air medical critical care transport services in the southern USA with coverage across two states. Their air medical patient volume exceeds 2200 annually and employs approximately 170 clinicians who work 12-hour shifts. Our third study site provides ground-based and air medical critical care services primarily in one state in the midwestern USA. Their patient volume is approximately 7000 annually. The system employs 150 clinicians who work full-time or part-time on 12-hour or 24-hour shifts. Study site 4 is located in the northeastern USA and reports an annual patient volume of more than 1100. The site employs approximately 55 air medical clinicians. All study sites hire clinicians with multiple years of clinical experience in advanced life support EMS systems or in critical care settings. Sites require clinicians to maintain advanced levels of certification and be licensed or certified as a flight paramedic, flight nurse, or respiratory therapist. All sites deploy clinicians in teams of two (dyads) as paramedic-paramedic, paramedic-flight nurse, flight nurse-flight nurse, or flight nurse-respiratory therapist teams.

### Recruitment and enrollment protocol

We will begin recruitment by using email to distribute a study flyer to eligible air medical clinicians affiliated with our four study sites. Clinicians with interest in the study are directed to contact site coordinators for screening. Eligible clinicians complete the following steps: (1) text a unique statement to the study’s telephone number to register his/her cell/smartphone with our automated texting system; (2) visit the study’s designated and password-protected webpage to watch an Institutional Review Board (IRB)-approved informed consent video; (3) indicate a decision to voluntarily participate or not by clicking ACCEPT or DO NOT ACCEPT. The time of an ACCEPT decision is defined as the time of enrollment; (4) if the clinician agrees to participate, he/she will create a unique login/password to use with the study website; (5) the participant will then login to the study website and answer a set of standard baseline demographic, sleep, fatigue, and shift work questions; and (6) use a calendar feature on the study website to document his/her shift work schedule for the next 120 days (4 months). We will encourage adherence with study procedures and assessments by (1) emphasizing at enrollment that participants try their best to answer all text-message assessments; (2) asking participants do their best to keep up with their shift schedule calendar via the study website; and (3) by sending automated reminder text messages to participants at the end of inter-shift assessments that emphasize the importance of maintaining an accurate shift schedule on the study website.

We will invite a convenience sample of 20 participants enrolled in the study to wear wrist actigraphs and complete Psychomotor Vigilance Tests (PVTs) at the start, midpoint, and end of select scheduled shifts. Wrist actigraphy is commonly used to provide objective measures of sleep patterns, and the PVT is a standard measure of cognitive performance [15, 16]. Data from the sample of 20 will be used to corroborate self-reported sleep and compare against self-reported fatigue, sleepiness, and difficulty with concentration taken during shift work.

### Participants

Participants must meet the following inclusion criteria: (1) be 18 years of age or older at time of enrollment; (2) work at one of the four study sites as an air medical clinician; (3) currently work shifts; (4) have a text-message (short message service, SMS)-capable cellphone/smartphone; and (5) be willing to take part in a research study that requires the sending and receiving of text messages daily over a 120-day (4-month) study period. Participants may discontinue participation at any time by contacting the study staff and texting the word “STOP” to the study phone number.

### Randomization

Randomization occurs at the individual level with 1:1 allocation and is executed when the participant completes step 1 of the enrollment protocol described above. Allocation to intervention or control is assigned in real time using a Structured Query Language (SQL) server procedure code imbedded in the web-based data collection system. Our algorithm restricts the cumulative sample size imbalance to no more than four participants between groups.

### Intervention

**Fig. 2:**
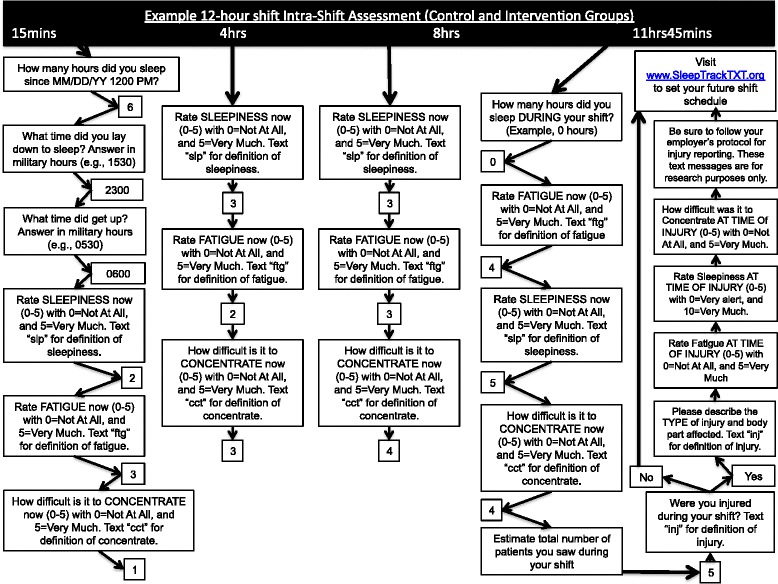
Flow diagram of intra-shift text-message assessments for intervention and control participants

**Fig. 3:**
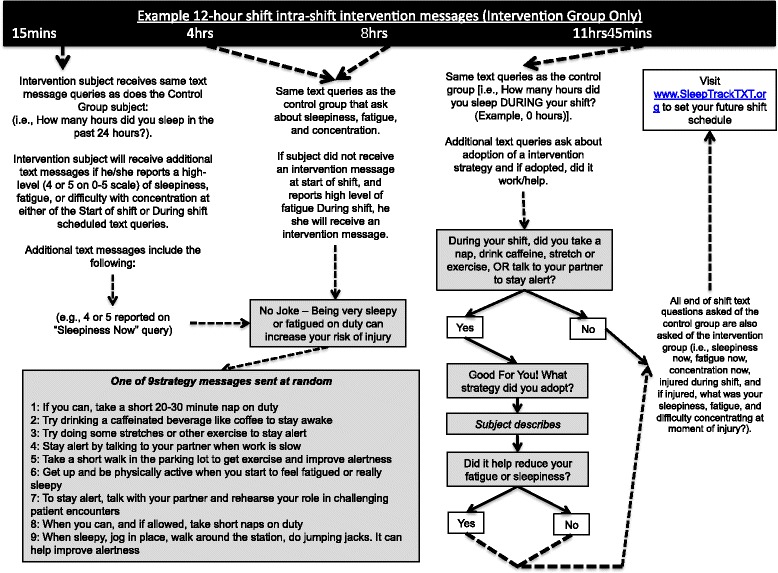
Flow diagram of intra-shift text messages sent to intervention participants prompting behavior change to improve alertness during shift work

**Fig. 4:**
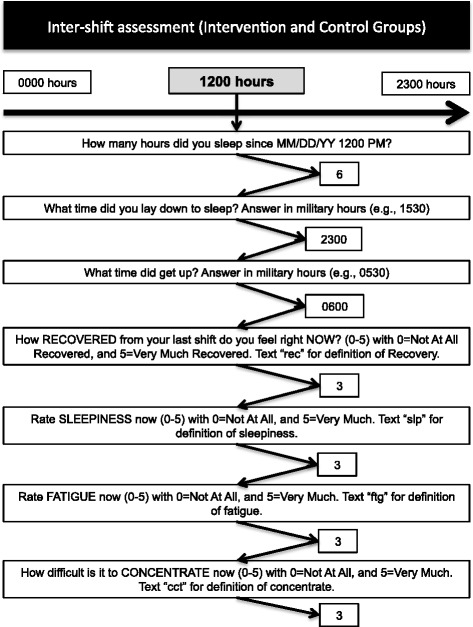
Flow diagram of inter-shift text-message assessments for both intervention and control participants

**Fig. 5:**
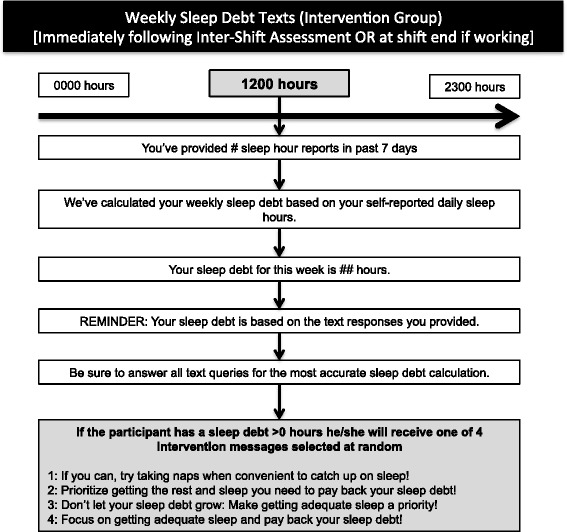
Flow diagram of sleep debt-related text messages sent on Friday of each week to intervention participants

Our non-pharmacological intervention is scheduled for 4 months (120 days) and incorporates elements of the original SleepTrackTXT intervention [7, 9], which include automated text messages promoting alertness behaviors during shift work for those participants reporting high levels of fatigue, sleepiness, or difficulty with concentration. These messages encourage a clinician to adopt alertness-promoting behaviors suggested in prior research as helpful with decreasing perceived fatigue and/or sleepiness [17–27]. Our prior publications of the SleepTrackTXT intervention highlight the content of four messages [7, 9]. We have added five additional intervention messages that encourage behavior change during shift work. Figures 2, 3, 4, and 5 provide a visual illustration and flowchart of text messages for participants in the control and intervention groups and include the details of our nine intervention messages that encourage adoption of alertness-promoting behaviors (see Fig. 3 for details). Intervention participants will receive additional text messages on Friday of each week summarizing their sleep debt over the previous 7 days (see Fig. 5 for details). The sleep debt calculation is computed automatically by summing daily sleep hours, then subtracting from 49, the minimum number of sleep hours a person should achieve over a one-week period per recommendations from the National Sleep Foundation. Following notice of a sleep debt, the participant receives a text message that encourages paying back the sleep debt when convenient (Fig. 5).

### Control

Participants randomized to the control group will not receive recommendations for improving alertness during shift work or for repaying weekly sleep debt (Fig. 2). Control subjects will receive text messages for assessment only at the beginning, during, and end of shifts for 120 days.

### Remuneration

All participants receive $20 at baseline enrollment and $20 each month of the study for a total of $100. Our remuneration is based on success with our prior research [7, 9].

### Measures

#### Participant-level measures

Following informed consent, participants will answer a standard demographic survey. Other baseline questionnaires will include the Pittsburgh Sleep Quality Index (PSQI), Chalder Fatigue Questionnaire (CFQ), select items from the Schedule Attitudes Survey (SAS), the Epworth Sleepiness Scale (ESS), the Occupational Fatigue Exhaustion Recovery (OFER) scale — with permission from the developer — and the Sleep, Fatigue, and Alertness Behavior (SFAB) survey. The PSQI is a widely used measure of sleep quality [28]. A PSQI score ≥6 on a 0–21 scale indicates poor sleep quality [28]. The CFQ is an 11-item measurement of mental and physical fatigue that has two scoring options [29]. Severe mental and physical fatigue is indicated on the CFQ score if the total score exceeds one of two scoring benchmarks. The SAS is a 21-item scale that measures worker attitudes pertaining to his/her shift schedule [30]. Higher scores on SAS items signify positive views towards a shift schedule. The ESS is widely used to measure daytime sleepiness [31]. The ESS scores range from 0 to 24, where scores 0–7 indicate unlikely abnormal sleepiness, 8–9 average sleepiness, 10–15 situational sleepiness, and ≥16 indicate excessive daytime sleepiness [31]. The OFER is a 15-item scale that measures acute fatigue, chronic fatigue, and inter-shift recovery [32]. Higher OFER scores (≥80) indicate more acute or chronic fatigue and less recovery between shifts. The SFAB survey is a construct valid tool comprising 50 items and 8 domains that measure behavioral intent to improve alertness during shift work [33]. Higher SFAB scores indicate a positive intent to adopt behaviors that may improve alertness while working shifts. All participant-level measures (survey and text-message data) will be maintained on a secure SQL database maintained by the coordinating site. Data quality is evaluated in real time with automated checks on acceptable and unacceptable values from participants. Participants who submit an unacceptable value are notified immediately with an error message and asked to reply with a correct value/answer. Actigraph and PVT data will be collected using IRB-approved electronic devices, assigned non-specific code numbers, stored securely at study sites, and supplied to the coordinating center using IRB-approved file transfer systems.

#### Shift-level measures

We will use single-item measures of clinician fatigue, sleepiness, and difficulty with concentration distributed to participants at the start, every 4 hours during, and at the end of scheduled shifts (see Fig. 2) [7, 9]. We have added additional single-item assessments to occur between scheduled shifts (inter-shift). See Fig. 4. The inter-shift assessments will include assessments of fatigue, sleepiness, and difficulty with concentration, as well as an item designed to capture perceived recovery since the last shift worked. Single-item measurement was used in the original SleepTrackTXT trial; it is favored for research that incorporates real-time data capture and is based on principles of ecological momentary assessment (EMA) [7, 9, 34, 35]. We hypothesize that, relative to participants in the control group, participants in the intervention group will report lower levels of fatigue, sleepiness, difficulty with concentration at the end of shift, and better levels of recovery between shifts.

#### Follow-up measures

**Fig. 6:**
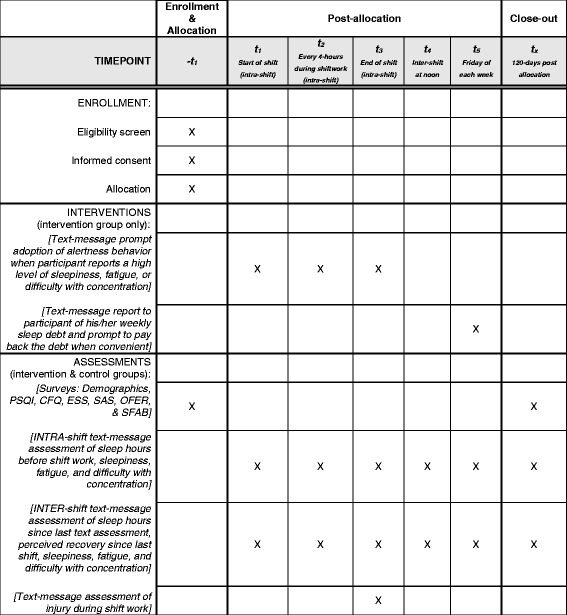
Schedule of enrollment, interventions, and assessments

Each study subject will complete the following survey assessment tools at 120 days and no longer than 150 days from baseline: the PSQI, CFQ, SAS, OFER, and SFAB. We report our full schedule of enrollment, intervention, and assessment activities in accordance with the SPIRIT checklist (see Fig. 6). The Principal Investigator will report the study’s progress, protocol modifications, any unanticipated events, or protocol deviations to the approving IRB bodies and to the University of Pittsburgh, Department of Emergency Medicine’s monthly Departmental Clinical Research Meeting (DCRM). Investigators, clinical faculty, and senior leaders of the university, who are independent of the study’s sponsor, attend this meeting. Audits will be requested and led by the approving IRB committees.

#### Sample size

Sleep quality as measured by the PSQI is our primary endpoint, and intra-shift fatigue is our secondary endpoint of interest. We based our power calculation on detecting a clinically meaningful difference of 3 points on the PSQI from baseline to the study end. The standard deviation of the difference in sleep quality was estimated as 2.2 using data from the original SleepTrackTXT randomized trial [7]. With group sample sizes of 23 we will achieve 90% power to reject the null hypothesis of equal means, conservatively assuming a larger standard deviation of 3 but same difference between groups and a two-sided two-sample equal variance *t* test (α = 0.05). Our estimate of attrition is 15%, based on our prior randomized trial, which inflates the sample size to 27 per group [7]. We further estimate that 15% of participants in the intervention group will not abide by the intervention protocol. Given these estimates, we seek to enroll a total of *n* = 100 (50 per group) to provide a robust number if the attrition and non-compliance were underestimated.

#### Statistical analysis

We will use the intention-to-treat principle for all analyses. Descriptive statistics will be calculated by group to assess the effectiveness of the randomization. Any clinically meaningful imbalances will be controlled for in sensitivity analysis secondary to the primary analyses proposed. For our primary outcome of sleep quality at 4 months (PSQI), we will test for the intervention effect using an analysis of covariance controlling for baseline sleep quality. For the secondary outcome of fatigue (measured multiple times per shift per person), we will use linear mixed model analyses to examine the effectiveness of the intervention on reducing within-shift fatigue. We will construct a model where the outcome is the change in fatigue from the beginning of the shift to the end. Our model will include a fixed effect for the experimental group, a fixed effect of time in study, and a random effect for subjects to account for multiple shifts within each person. From the model, we will examine the estimated change in fatigue score per shift in the intervention group compared to that in the control group. The same methodology will be used to analyze end-of-shift sleepiness and difficulty in concentrating.

For all tertiary outcomes (SAS, OFER, SFAB), we will test for between-group differences in the scales using analysis of covariance with a fixed effect for group controlling for the baseline measures assuming the underlying distributions are normal. The CFQ will be dichotomized at a score of ≥4 and compared using Fisher’s exact test. If the data are highly skewed, we will use non-parametric tests (Mann-Whitney *U* tests). All statistical testing will be two-sided (α = 0.05), performed using SAS version 9.2 (SAS Institute Inc., Cary, NC, USA).

## Discussion

Findings from the original SleepTrackTXT trial (ClinicalTrials.gov NCT02063737) demonstrated that use of mobile phone text messages could play an important role in real-time assessment and intervention of EMS clinician fatigue [7]. The SleepTrackTXT2 study will test an enhanced intervention and assess short-term impact on fatigue and long-term impact on clinician sleep quality. The current trial will provide much-needed data to guide development of novel fatigue risk management programs tailored to air medical EMS clinicians. Others may choose to replicate our protocol in different clinician groups or shift worker populations where real-time fatigue risk management may be needed.

The principal strength of our study is that it has been designed based on the recently successful SleepTrackTXT trial [7]. That study had many successes, which make modeling a new protocol appropriate. Compliance with text-message assessments was high (88%). Participants answered 36,073 of 40,947 text-message assessments. Attrition was low (15%), and at the study’s conclusion, 95% (*n* = 82) of participants reported a willingness to answer text-message assessments on days with and without scheduled work periods.

One innovation of the current study protocol (SleepTrackTXT2) is the addition of an inter-shift assessment of sleep hours, recovery since last shift worked, fatigue, sleepiness, and difficulty with concentration. Daily assessments, rather than shift-only assessments, will permit us to fill gaps in our understanding of EMS clinician sleep habits before, during, and in-between scheduled shifts. We also added an assessment of perceived inter-shift recovery. Recovery is described as an improvement in a worker’s perceived mental and/or physical fatigue after a period of rest between periods of work [36–38]. Prior studies show that a worker needs a minimum of 16 hours of free time, and likely more, in order to attain 7–8 hours of sleep [39]. Our recent study of *n* = 450 EMS clinicians who answered a reliable and valid questionnaire of inter-shift recovery showed that half do not get the recovery they need between scheduled shifts [8]. The addition of a recovery assessment will permit us to determine the mean time to full recovery post-shift work specific to our target population.

Another innovation of this study is the weekly reporting of sleep debt for the intervention group. Sleep debt refers to “the cumulative hours of sleep loss with respect to a subject-specific need for sleep” [40]. The National Sleep Foundation recommends that adults achieve between 7 and 9 hours of sleep in a 24-hour period to achieve optimal rest and sleep-associated health benefits. Prior population-level investigations of sleep have historically used total daily sleep hours <7 hours as a benchmark for inadequate sleep [41]. Sleep debt is a public health problem for the US adult working population. A recent Behavioral Risk Factor Surveillance System survey showed that more than 35% of the US adult population reported <7 hours of sleep in the previous 24 hours [41]. Findings from a recent National Health Interview Survey show that 30% of US adults report ≤6 hours of sleep daily [42]. Sleep debt or sleep restriction over a matter of hours or days has been linked to reduced cognitive performance, reduced daytime alertness, disruption of normal metabolic functioning, and disordered regulation of hormone secretion, heart rate, and blood pressure, which can lead to cardiovascular disease [43–47]. Our intervention is novel because it calculates sleep debt on a weekly basis, reports this debt to the intervention participant each week during the study period, and prompts the participant to “pay back” the sleep debt with rest, sleep, and recovery. We believe that this component of the intervention is meaningful and may have the greatest potential for impact, given the persistent lack of adequate sleep reported by EMS clinicians [48].

Finally, findings from this study will offer preliminary data for further honing the details of fatigue risk management programs and interventions specifically for EMS clinicians. This is the second study of its kind to use mobile phone text messages as a vehicle to conduct real-time assessment and intervention of a unique clinician population [7, 9].

Our study faces several potential challenges. In particular, we are attentive to the volume of text messages in this study. Compared to the original SleepTrackTXT study, the current study includes more text-message queries, which may or may not be well received by participants. Previous research has suggested that the ideal number of text messages per day is 5.5 (range 1–20) [49]. Participants in the original SleepTrackTXT trial reported a willingness to answer text-message queries on days off from work (inter-shift) as well as during work [7]. Our current protocol is based on these observations and lessons learned from prior research. However, it is unclear if participants of the current study will perceive the volume of text messages as reasonable or bothersome. Given the built-in differences in volume of text messages, attrition may be higher in the intervention versus the control group. We have included a modest incentive for participants to address the threat of attrition. We do not believe participant performance, attrition, or response to text-message queries will be affected by group status. Participants are informed that the study involves two groups during the consent process. However, participants are not informed of group status post-randomization.

Fatigue in the air medical emergency care setting is a threat to patient and clinician safety. We propose an innovative use of mobile technology to reduce perceived fatigue and mitigate its impact in a high-risk setting. The SleepTrackTXT2 trial may provide evidence of real-world effectiveness that would support widespread expansion of real-time fatigue mitigation interventions in emergency care clinician shift workers.

### Trial status

The date of first enrollment was 6 June 2016. The date of last enrollment was 23 March 2017. Participants were no longer being recruited or enrolled as of 31 March 2017. Data collection of the remaining participants was ongoing as of 10 May 2017.

## Additional file

Additional file 1: SPIRIT 2013 checklist: recommended items to address in a clinical trial protocol and related documents. (DOCX 65 kb)

## Abbreviations

CFQ: Chalder Fatigue Questionnaire
ED: Emergency department
EMA: Ecological momentary assessment
EMS: Emergency Medical Services
mHealth: Mobile health
OFER: Occupational Fatigue Exhaustion Recovery (scale)
PSQI: Pittsburgh Sleep Quality Index
SAS: Schedule Attitudes Survey
SFAB: Sleep, Fatigue, and Alertness Behavior (survey)
SMS: Short message service
US: USA, United States
